# Upper-Limb Disability and the Severity of Lymphedema Reduce the Quality of Life of Patients with Breast Cancer-Related Lymphedema

**DOI:** 10.3390/curroncol30090585

**Published:** 2023-08-31

**Authors:** Karol Ramirez-Parada, Angela Gonzalez-Santos, Layla Riady-Aleuy, Mauricio P. Pinto, Carolina Ibañez, Tomas Merino, Francisco Acevedo, Benjamin Walbaum, Rodrigo Fernández-Verdejo, Cesar Sanchez

**Affiliations:** 1 Department of Health Sciences, School of Medicine, Pontificia Universidad Católica de Chile, Santiago 8330077, Chile; kramirezp@uc.cl; 2 Department of Physical Therapy, Faculty of Health Sciences, University of Granada, 18071 Granada, Spain; angelagonzalez@ugr.es; 3 Biosanitary Research Institute of Granada—Instituto de Investigación Biosanitaria ibs.GRANADA, 18012 Granada, Spain; 4 ’Cuídate’ from Biomedical Group (BIO277), Instituto de Investigación Biosanitaria ibs.GRANADA, University of Granada, 18071 Granada, Spain; 5 Department of Lymphatic Rehabilitation and Esthetics, Lymphology Clinic, Santiago 7510032, Chile; 6 Support Team for Oncological Research and Medicine (STORM), Santiago 8330077, Chile; mauricio.pinto@usach.cl; 7 Departament of Hematology and Oncology, School of Medicine, Pontificia Universidad Católica de Chile, Santiago 8330077, Chile; caibanez@uc.cl (C.I.); trmerino@uc.cl (T.M.); fnaceved@uc.cl (F.A.); bvwalbau@uc.cl (B.W.); 8 Laboratorio de Fisiología del Ejercicio y Metabolismo (LABFEM), Escuela de Kinesiología, Facultad de Medicina, Universidad Finis Terrae, Santiago 7500000, Chile

**Keywords:** breast neoplasms, disability evaluation, breast cancer-related lymphedema

## Abstract

Breast cancer-related lymphedema (BCRL) is characterized by arm swelling, pain, and discomfort, reducing the quality of life (QoL) of affected individuals. BRCL is caused via the blockage or disruption of the lymphatic vessels following cancer treatments, leading to an accumulation of fluid in the affected arm. While current BCRL rehabilitation treatments seek to reduce arm swelling, our study aimed to examine the impact of both the magnitude of lymphedema (ΔVolume) and arm disability on three dimensions of QoL: social, physical, and psychological. Using the Disabilities of the Arm, Shoulder, and Hand questionnaire (DASH) and the Upper Limb Lymphedema 27 questionnaire (ULL) in a group of 30 patients, we found that the magnitude of lymphedema (ΔVolume) was associated with the social dimension of QoL (*r* = 0.37, *p* = 0.041), but not with other dimensions. On the other hand, arm disability was associated with all evaluated dimensions of QoL (social, physical, and psychological: *p* < 0.001, *p* = 0.019, and *p* = 0.050 (borderline), respectively). These findings suggest that BCRL rehabilitation strategies should not only aim to reduce the magnitude of lymphedema but should also seek to improve or preserve arm functionality to enhance the QoL of BCRL patients.

## 1. Introduction

In recent decades, major advances in the diagnosis and treatment of breast cancer (BC) have translated into an increase in long-term patient survival [1,2]. Unfortunately, this gain in survival is also accompanied via an increase in the incidence of long-term post-treatment complications [3,4,5]. Among these, BC-related lymphedema (BCRL) is a frequent treatment-derived complication [6,7]. Lymphedema in these patients is caused via the obstruction or disruption of the lymphatic system [7]. In the absence of adequate drainage by lymphatic vessels, protein-rich lymphatic fluid accumulates in the interstitial space causing abnormal swelling of the affected side (edema) that may compromise the breast, trunk, and/or the upper limb [8]. Several studies indicate a 10–40% incidence of BCRL after regional nodal irradiation and 10–50% following axillary dissection [6,9,10,11,12,13]. Some symptoms of BCRL may include arm stiffness, numbness, heaviness, pain, and decreased functioning of the upper limb [14]. Evidently, all these symptoms are associated with a reduction in patients’ quality of life (QoL) with psychosocial consequences [15]. Indeed, BCRL is commonly associated with depression, anxiety, distress, and irritation [15,16].

Physical disability among BCRL patients results from the reduction in the range of motion of the shoulder and arm [17,18], while psychological disturbances including distress, depression, irritation, and social limitations result from difficulties to perform daily activities [15]. To date, the most widely accepted conservative treatment strategy for BCRL is the complex decongestive therapy (CDT) that seeks to reduce the volume of the affected arm by combining manual lymphatic drainage (MLD), exercise, and compression bandages [19]. Surgical procedures like lymphaticovenular anastomosis (LVA) or vascularized lymph node transfer (VLNT) can also be considered as a second-line alternative for patients when CDT becomes ineffective [20]. Unfortunately, the effectiveness of CDT is still uncertain [21]. Similarly, there is no current agreement on the timing, staging, indication or the potential combination of surgical procedures, and therefore their true efficacy cannot be reliably assessed [20]. Overall, current recommendations call for a more comprehensive, integrated, multidisciplinary treatment for BCRL, with emphasis on the rehabilitation of patients and their QoL [22].

As pointed earlier, it is well established that BCRL can lead to a reduction in patients’ QoL [9,16,17]. However, the specific contributions of both the severity of BCRL and the upper-limb disability over different dimensions of QoL are still undetermined [6,9,10,15,17,23]. Therefore, our study sought to determine the association between the severity of BCRL/upper-limb disability and the physical, psychological, and social dimensions of QoL in BC female patients affected by BCRL.

## 2. Materials and Methods

### 2.1. Study Design and Sample

This was an observational, cross-sectional study. A convenience sample was used, considering all women referred to the Oncology Physical Therapy Service at the Complejo Asistencial Dr. Sótero del Río in Santiago, Chile, with BCRL diagnosis. The data were collected between July and September 2019. The study was conducted in compliance with the Declaration of Helsinki, and the Ethical Board at South-East Metropolitan Health Service approved the study. All participants provided written informed consent before entering the study.

### 2.2. Patient Eligibility

Our study included adult (≥18 year-old) female BC patients with confirmed BCRL, without previous treatments for lymphedema. Age and BMI were registered at the Oncology Physical Therapy Service. Other information was obtained from medical records, including number of removed lymph nodes, type of breast and axillary surgery, use of neoadjuvant or adjuvant chemotherapy and radiotherapy, and radiotherapy field (breast/chest wall or including lymph node basin).

### 2.3. BCRL Diagnosis and Severity

An experienced physiotherapist conducted assessments (K.R.-P.). The criteria for BCRL [9] diagnosis were: (a) having >10% of volume difference between the affected and the contralateral upper limb; (b) having >200 mL of volume difference between the affected and the contralateral upper limb; or (c) reporting arm tightness or heaviness/fullness in the affected upper limb. The measurement of the upper-limb volume was conducted with an optoelectrical volumetry perometer, as described by [24]. Briefly, patients were positioned facing the perometer while placing the limb at 90 degrees relative to the trunk (Supplementary Figure S1). Then, the perometer measured the upper-limb diameter every 4.7 mm to calculate the overall upper-limb volume. The magnitude of BCRL was defined as the difference in volume (ΔVolume in mL) between the affected and the contralateral upper limb.

BCRL can be categorized into stages based on the severity of the condition [25]. Stage 0: Subclinical Stage: the patient is considered “at-risk” for lymphedema development due to injury to the lymphatic vessels but does not present with outward signs of edema. Stage 1: Mild lymphedema: the swelling is mild and usually reversible with elevation and rest. Stage 2: Moderate lymphedema: involves moderate swelling that does not reduce significantly with limb elevation. Tissue fibrosis might begin to develop, causing a harder texture in the affected area. Stage 3: Severe lymphedema: the swelling is severe and does not reduce with elevation. The skin becomes thickened, and there may be extensive tissue fibrosis. Infections are more likely to occur, and the upper limb can become immobile (disability).

### 2.4. Upper-Limb Disability and QoL Assessment

The same physiotherapist conduced these assessments (K.R-P). Upper-limb disability was assessed using the Disabilities of the Arm, Shoulder, and Hand (DASH) questionnaire that includes 30 questions evaluating the difficulty of performing daily activities. Each question has a score from one to five, where one represents “no difficulty” and five represents “impossible to complete”. The final score is calculated as: DASH score = [(sum of responses) − 30]/1.2. Scores range from 0 (no disability) to 100 (most severe disability). [26]. The Spanish version of the DASH questionnaire has good reliability, stability, and responsiveness to change [27]. Also, the DASH questionnaire has been used in BC patients [28].

Grip strength was measured on a hand dynamometer (Jamar). The maneuver was conducted three times, with a 1 min rest between attempts. We used the best result. Both hands (affected arm and non-affected arm) were measured and compared. The results were expressed in kilograms. Intra-instrument reliability and concurrent validity were tested using certified standard weights (r = 1.00), while inter-instrument reliability was good between 0.80 and 0.83 [29]. Further, there are reference values for a healthy Chilean population [30,31].

The pain intensity was assessed on a 100 mm visual analog scale, where scores ranging from 0 to 4 mm corresponded to no pain; 5 to 44 mm, mild pain; 45 to 74 mm, moderate pain; and 75 to 100 mm, severe pain [32]. The visual analog scale has been shown to be valid for measuring postoperative pain in patients with BC [33].

Next, QoL was evaluated using the Upper Limb Lymphedema (ULL) 27 questionnaire. The ULL includes 27 items to assess the physical (15 items), psychological (7 items), and social (5 items) dimensions of QoL. Scores range from 0 to 100. Higher values indicate poorer quality of life [34]. The Spanish version of the ULL 27 has been proven valid and reliable to assess the QoL of patients with lymphedema [35].

### 2.5. Statistical Analysis

The data are presented as mean [standard deviations] or percentages. Both BMI and ΔVolume were non-normally distributed (Shapiro–Wilk test) and were thus log_10_-transformed prior to the analyses. Associations between continuous variables were tested with Pearson’s *r* test. Stepwise multiple linear regression was used to identify the predictors of physical, social, and psychological ULL dimensions. DASH score, ΔVolume, age, and BMI were included as candidate predictors. Fisher’s exact test was used to compare the frequencies of categorical variables between those groups. IBM^®^ SPSS^®^ Statistics version 26 was used for the analyses. Statistical significance was set at *p* < 0.05. All data were blindly analyzed by a statistician.

## 3. Results

A total of thirty female BC patients diagnosed with BCRL were included in our study. Patients’ basic clinical characteristics are summarized in Table 1. Briefly, patients had predominantly stage III BC (>50%). Most patients underwent axillary lymph node dissection (96.7%) and received radiotherapy (96.7%). Notably, 28 of them (93%) were overweight or obese (BMI ≥ 25 kg/m^2^).

**Figure 1:**
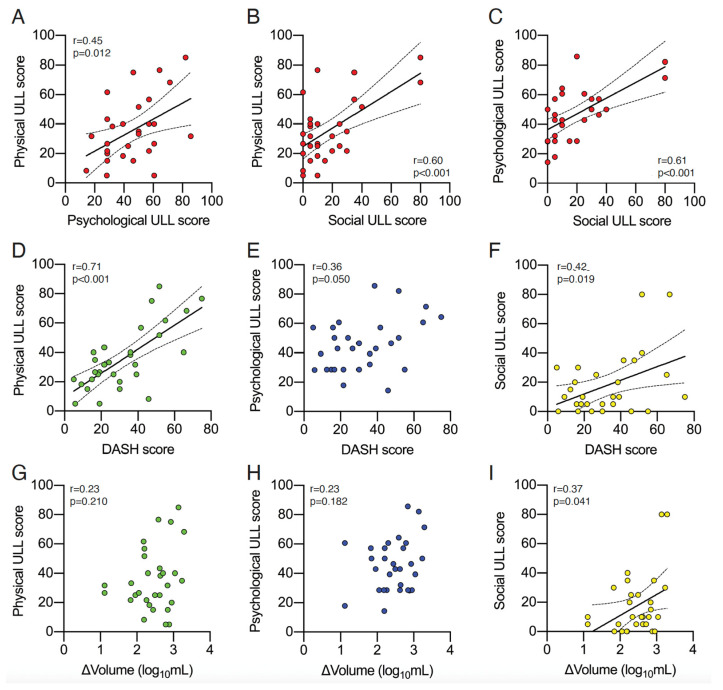
Consistency of ULL dimensions and their association with DASH scores and ΔVolume in BCRL patients. (**A**–**C**). The solid lines indicate the linear regression, and the dotted lines indicate the 95% confidence intervals: (*n* = 30). (**D**–**I**). Associations between physical ULL (green dots), psychological ULL (blue dots), and social ULL (yellow dots) dimensions, and the disabilities of the arm, shoulder, and hand (DASH). (**D**–**F**) The questionnaire or ΔVolume. (**G**–**I**). The solid lines indicate linear regression, and the dotted lines indicate the 95% confidence interval: (*n* = 30).

First, we sought to determine if the measured ULL scores were consistent across all dimensions (physical, social, and psychological). Figure 1A–C shows that all dimensions were directly associated with each other. Next, we evaluated potential associations between QoL/ULL dimensions and DASH scores (upper-limb disability) or between ULL and ΔVolume (severity of lymphedema). We found that the physical ULL scores were associated with DASH scores (Figure 1D), but not with ΔVolume (Figure 1G). Similarly, we found a borderline significant association between psychological ULL scores and DASH scores (*p* = 0.05), but not with ΔVolume (Figures 1E,H, respectively). Social ULL scores were directly associated with both DASH scores and ΔVolume (Figure 1F,I). Notably, ΔVolume (i.e., severity of lymphedema) and DASH scores (upper-limb disability) were not associated (Pearson *r* = 0.23, *p* = 0.21, *n* = 30).

Finally, we searched for potential predictors for the different dimensions of QoL/ULL and conducted a series of stepwise multiple linear regressions. Table 2 shows that DASH scores, age, and ΔVolume explained 63% of the variance in the physical ULL. As for the psychological ULL, the DASH score was a borderline (*p* = 0.05) predictor in a model that explained only 9% of the variance. Lastly, the DASH score was the unique predictor for the social ULL, explaining 15% of the variance.

## 4. Discussion

In recent decades, the development of novel, more effective, and multimodal treatment strategies against BC has been translated into an increase in the burden of cancer survivors [1]. This phenomenon has focused the interest of clinicians in the QoL of cancer survivors and its association with treatment-derived complications. Our study sought to determine if the severity (or magnitude) of BCRL or the upper-limb disability had an impact upon the different dimensions of BC patients’ QoL. Our results suggest that more severe BCRL is associated with a poorer social dimension in QoL. This factor also explains 8% of the variance in the physical dimension of QoL. Similarly, higher levels of upper-limb disability are associated with poorer social and physical dimensions of patients’ QoL. We also found a borderline association between upper-limb disability and the psychological dimension. Upper-limb disability explains 49%, 15%, and 9% of the variance in the physical, social, and psychological dimensions, respectively. In general, BCRL patients with lower levels of QoL are characterized by a higher upper-limb disability and higher pain. It is noteworthy that most patients in our cohort (93%) were either overweight or obese. Although several BCRL-risk factors have been postulated throughout the literature, including excess body weight (BMI ≥ 25 kg/m^2^), delayed wound closure, postoperative infections, hypertension, and taxanes chemotherapy [12], their definitive contribution to the development of BCRL and a precise mechanism remains undefined.

Based on our findings, we hypothesize that maintaining upper-limb functionality, rather than the severity of BCRL, is a key factor to improve survivors’ QoL. Indeed, upper-limb disability seems to be more relevant for QoL versus the magnitude or severity of BCRL. This is in line with a previous study by Bojinović-Rodić et al. [28] that demonstrates that upper-limb function is associated with the physical, emotional, and social dimensions of QoL, whereas the size of lymphedema remained unrelated. A similar study compared BC patients with or without BCRL and concluded that arm symptoms are more informative for QoL than arm swelling [36]. While most studies emphasize the association between arm disability and QoL, other variables such as the type of work and the level of physical activity of patients may also have an impact on this association. Future studies should determine and analyze the relevance of these variables.

Interestingly, we did not find an association between ΔVolume and the DASH score in our study, suggesting the severity of BCRL and upper-limb disability are unrelated. Accordingly, a previous study by Hayes et al. [37] found no association between the severity of lymphedema and upper body function in a cohort of 287 BC patients. Moreover, a prospective study by O’Toole et al. [38] demonstrated that changes in lymphedema volume and a clinically significant BCRL does not affect limb functionality. Therefore, based on these findings we speculate that upper-limb disability is an independent factor within the causal pathway between the severity of BCRL and QoL.

Besides upper-limb functionality, other predictors of the physical dimension of QoL in our study included age and volume difference. Again, these observations are in line with the findings by Zhang et al. [16] that included emotional distress as a predictor. A second study also demonstrated that an increase in upper-limb volume was a predictor of QoL in BC patients [23]. Notably, the questionnaire applied by the investigators in the abovementioned study did not discriminate between QoL dimensions. Overall, our data suggests that upper-limb volume mainly influences the physical dimension of QoL. In contrast, upper-limb functionality not only affects the physical dimension of QoL, but also acts as a predictor of psychological and social dimensions.

Pending further validation, our findings may have practical and clinical implications for the treatment of BCRL. While most current strategies seek to control the magnitude of BCRL by applying complex physical therapy, manual lymphatic drainage, laser therapy, pneumatic pump, compression bandaging, limb exercises and elevation, and even surgical procedures [20,39], our results point at functional improvement.

Studies demonstrate that >40% of the growing burden of cancer survivors suffer long-term consequences derived from the cancer itself and its treatment(s), which may include physical, cognitive, and psychological sequelae [40]. In line with current recommendations that point towards a more integrated, multidisciplinary approach to BC treatments that prioritizes QoL, our findings confirm the relevance of patient rehabilitation to preserve or improve limb functionality in BC survivors. Within this context, initiatives such as the ActivOnco model of care that promote active lifestyle and prescribed exercises can further improve the quality of survivorship [41] and should be encouraged for BCRL patients. Moreover, since BCRL is a chronic condition, we propose the inclusion of patient education programs to maintain arm functionality, perhaps adding self-management in clinical practice guidelines, and the inclusion of moderate-to-high intensity resistance exercise to improve functionality. Studies have demonstrated that this type of exercise is not only safe but also improves upper-limb function, prognosis, and QoL in BCRL patients [42,43]. Indeed, high physical activity levels are associated with better functionality in BCRL patients and is a predictor of upper-limb functionality [44]. In summary, the implementation of self-management programs to maintain/enhance limb functionality via physical activity (including exercise and daily activities) could benefit BCRL patients improving their QoL.

Our study has certain limitations. First, although we demonstrated an association between upper-limb disability and QoL, a causal relationship should be further investigated and validated by prospective studies in a larger cohort. Secondly, our small sample size may have precluded us from identifying associations between the severity of BCRL and other QoL dimensions. However, even if those associations existed, they would not affect the conclusions of our work that suggest implementing interventions with a special focus on the prevention or improvement of arm disability among BCRL patients.

In summary, given that lymphedema is a chronic condition, efforts should be focused on preventing its development. Our group promotes that exercise and an early and prospective physical therapy program can help prevent BCRL [45,46].

## 5. Conclusions

Upper-limb function is strongly associated with the QoL of patients that suffer BCRL. Therefore, BCRL treatments aiming to improve patients’ QoL should not only focus on reducing arm volume but should also prioritize the recovery of arm functionality.

## Figures and Tables

**Table 1:** Clinical characteristics of patients (*n* = 30).

Variable	Mean (SD) or %
Breast cancer stage	
IIA	13.3
IIB	13.3
IIIA	43.3
IIIB	6.7
IIIC	3.3
IV	20.0
Removed lymph nodes, *n*	17 (7)
Positive lymph nodes, *n* ^$^	3 (5)
Breast surgery	
Breast-conserving surgery	60.0
Breast ablation	40.0
Axillary surgery	
Axillary lymph node dissection	96.7
Sentinel lymph node biopsy	3.3
Adjuvant Radiotherapy	96.7
Lymph node basin Radiotherapy	100
Chemotherapy	
Adjuvant	50.0
Neoadjuvant	33.3
Hormone treatment	76.7
Stage of lymphedema	
I	30.0
II	43.3
III	26.7
Time with lymphedema	
<1 year	36.7
1 to 3 years	40.0
>3 years	23.3
∆Volume, mL	502 (499)
DASH, score	31.9 (18.8)
Dynamometry affected arm, kg ^$^	17.2 (6.0)
Dynamometry unaffected arm, kg ^$^	18.2 (5.4)
Pain, score ^&^	1.3 (2.3)
ULL, score	
Physical	35.6 (21.3)
Psychological	45.7 (17.8)
Social	17.5 (20.8)

^$^*n* = 27; ^&^ *n* = 28. Abbreviations: SD: standard deviation; ∆Volume: volume difference between affected arm versus contralateral arm; DASH: disabilities of the arm, shoulder, and hand; ULL: upper-limb lymphedema questionnaire.

**Table 2:** Predictive models of ULL from DASH, ∆Volume, age, and body mass index (*n* = 30).

ULL-27 Dimension	Predictors	Adjusted R^2^	β-Coefficient	*p*-Value	Regression Adjusted R^2^	Regression *p*-Value
Physical *	DASH	0.49	0.992	<0.001	0.63	<0.001
	Age	0.06	−0.787	0.002		
	∆Volume	0.08	14.477	0.018		
Psychological *	DASH	0.09	0.342	0.050	0.09	0.050
Social *	DASH	0.15	0.468	0.019	0.15	0.019

* Models were generated with a stepwise multiple linear regression analysis.

## Data Availability

The dataset generated and/or analyzed during the current study is available from the corresponding author upon reasonable request.

## Supplementary Materials

The following supporting information can be downloaded at: https://www.mdpi.com/article/10.3390/curroncol30090585/s1, Figure S1: Measurement of upper-limb volume using a perometer.
