# Supplementary figures and images for: Upper-Limb Disability and the Severity of Lymphedema Reduce the Quality of Life of Patients with Breast Cancer-Related Lymphedema
Source: Curr Oncol. 2023 Aug 31;30(9):8068–77. doi: 10.3390/curroncol30090585 (PMC10527643; doi:10.3390/curroncol30090585)

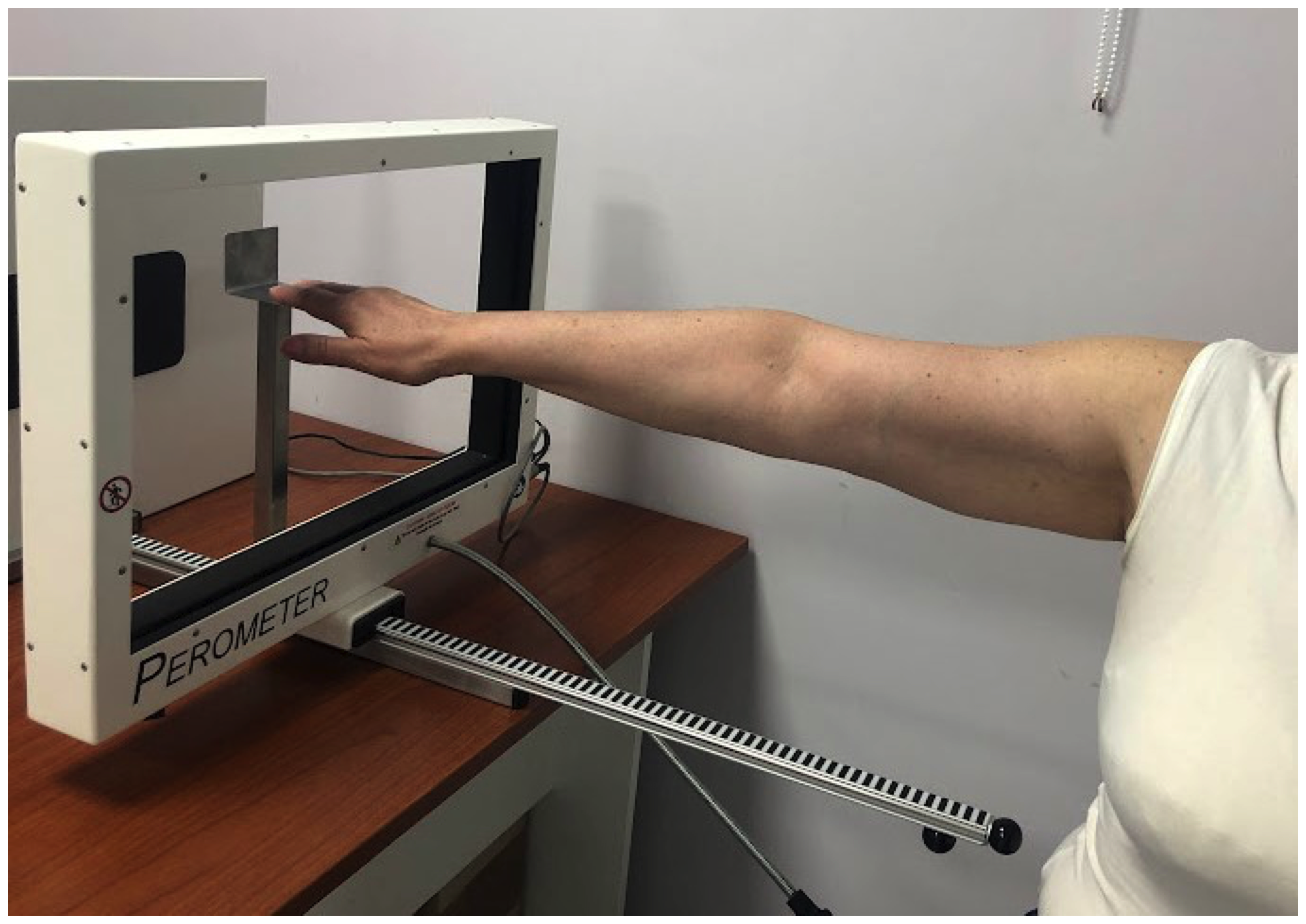

Supplement: Supplementary file 1 [file curroncol-30-00585-s001.zip › curroncol-2553259-supplementary.tiff]
